# Supplementary material for: Evaporation of a Sessile Colloidal Water–Glycerol Droplet: Marangoni Ring Formation
Source: Langmuir. 2022 Sep 12;38(39):12082–94. doi: 10.1021/acs.langmuir.2c01949 (PMC9536018; doi:10.1021/acs.langmuir.2c01949)
Supplement: Supplementary file 8 — la2c01949_si_008.pdf [file la2c01949_si_008.pdf]

Supporting Information: Evaporation of a Sessile Colloidal Water-Glycerol Droplet : Marangoni Ring Formation

Lijun Thayyil Raju,[†] Christian Diddens,[†] Yaxing Li,[‡] Alvaro Marin,[†] Marjolein N. van der Linden,[¶,†] Xuehua Zhang,[§,†] and Detlef Lohse[*,†,‖]

†*Physics of Fluids Group, Faculty of Science and Technology, University of Twente, 7500 AE Enschede, The Netherlands*

‡*Institute of Fluid Dynamics, Department of Mechanical and Process Engineering, ETH Zürich, 8092 Zürich, Switzerland*

¶*Canon Production Printing Netherlands B.V., 5900 MA Venlo, The Netherlands*

§*Department of Chemical and Materials Engineering, University of Alberta, Edmonton, Alberta T6G 1H9, Canada*

‖*Max Planck Institute for Dynamics and Self-Organisation, 37077 Göttingen, Germany*

E-mail: d.lohse@utwente.nl

Contents

S1 Experimental set-up **S-3**

S2 Contact line motion **S-4**

S3 Details of µPIV **S-4**

S4 Linearization coefficient in evaporation model **S-5**

S5 Marangoni ring for various initial compositions **S-6**

S6 Top-view visualization of particle-laden water droplet **S-7**

S7 Calculations of Rayleigh and Marangoni numbers **S-7**

S8 Additional data on location of Marangoni ring **S-8**

S9 Additional data on location of Marangoni vortex **S-9**

S10 Hypothetical influence of contaminants **S-9**

References **S-12**

S1 Experimental set-up

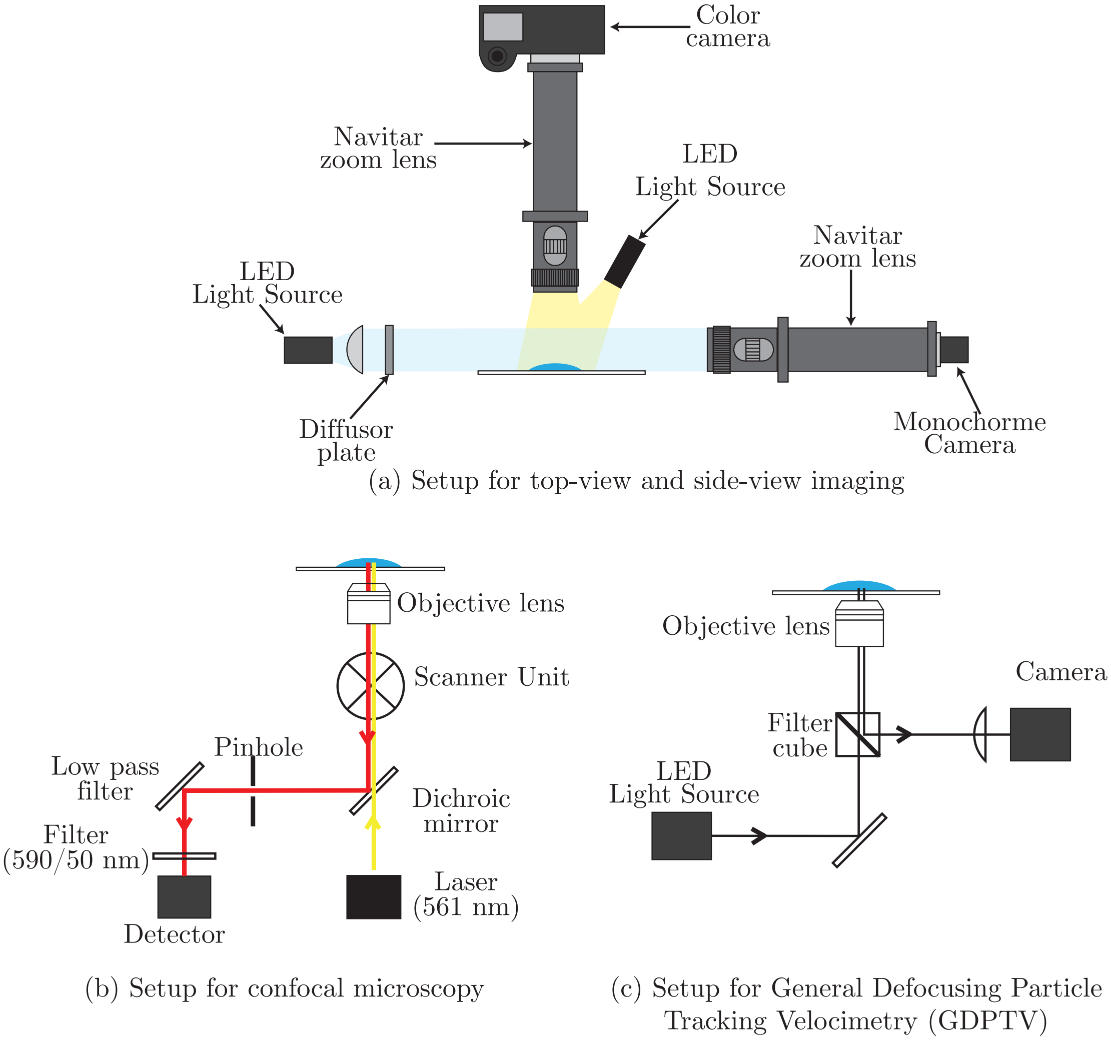

Figure S1: **Schematic diagram showing the various experimental set-up used in the study.** (a) Schematic representation of set-up used for top-view and side-view imaging of the droplet. (b) Simplified schematic representation of Nikon Confocal Microscope A1 plus system, based on the NIS-Elements AR software. (c) Schematic representation of an inverted microscope used for performing General Defocussing Particle Tracking Velocimetry (GDPTV).

S2 Contact line motion

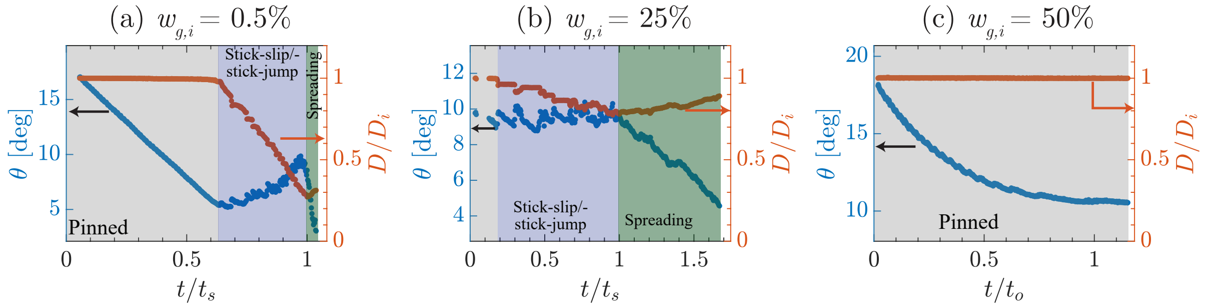

Figure S2: **Contact line dynamics** Plot showing variation of normalized footprint diameter (D/D_i) and apparent contact angle (θ) with normalized time, calculated from the side-view visualization of drop shape for droplets having different initial glycerol weight fractions ($w_{g,i}$) of (a) 0.5%, (b) 25% and (c) 50%. Similar to droplets with $w_{g,i} = 5\%$ (Figure 1c, main text), droplets with $w_{g,i} = 0.5\%$ and 25% also show the different modes of contact line motion, namely pinned, stick-slip, stick-jump, and spreading.

S3 Details of µPIV

Cross-correlations and the velocity field were obtained using PIVLab 2.53 (running in MATLAB). The following parameters were used in our case: FFT window deformation; first pass: interrogation area 64 pixels × 64 pixels with 50% overlap; second pass: interrogation area 32 pixels × 32 pixels with 50% overlap; sub-pixel estimator : Gauss 2×3 point; smooth data using the algorithm of Garcia D.[S1]

S4 Linearization coefficient in evaporation model

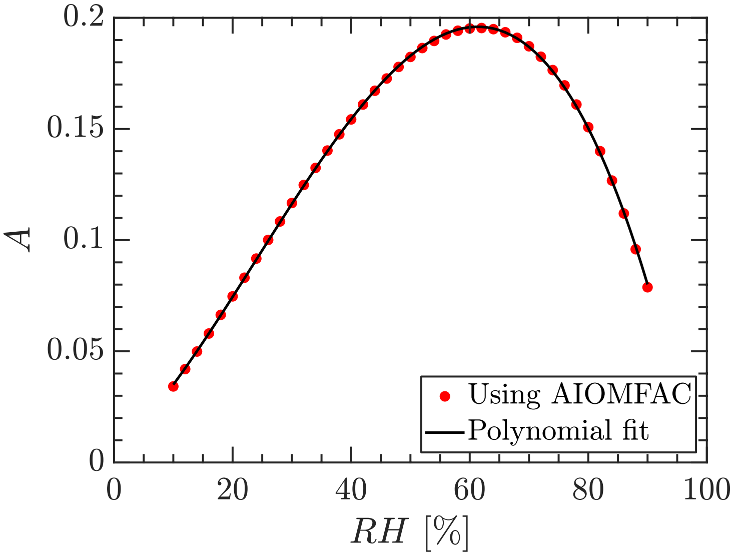

Figure S3: **Linearization coefficient (A) vs relative humidity (RH)**. Plot showing variation of A with RH at a temperature of 20 °C. The value of A is such that $c_{w,s} - c_{w,\infty}$ becomes zero at the same value of x_g as without any linearization. The continuous line shows a polynomial fit $A = -1.0016 \times RH^3 + 0.7186 \times RH^2 + 0.2492 \times RH + 0.0039$.

S5 Marangoni ring for various initial compositions

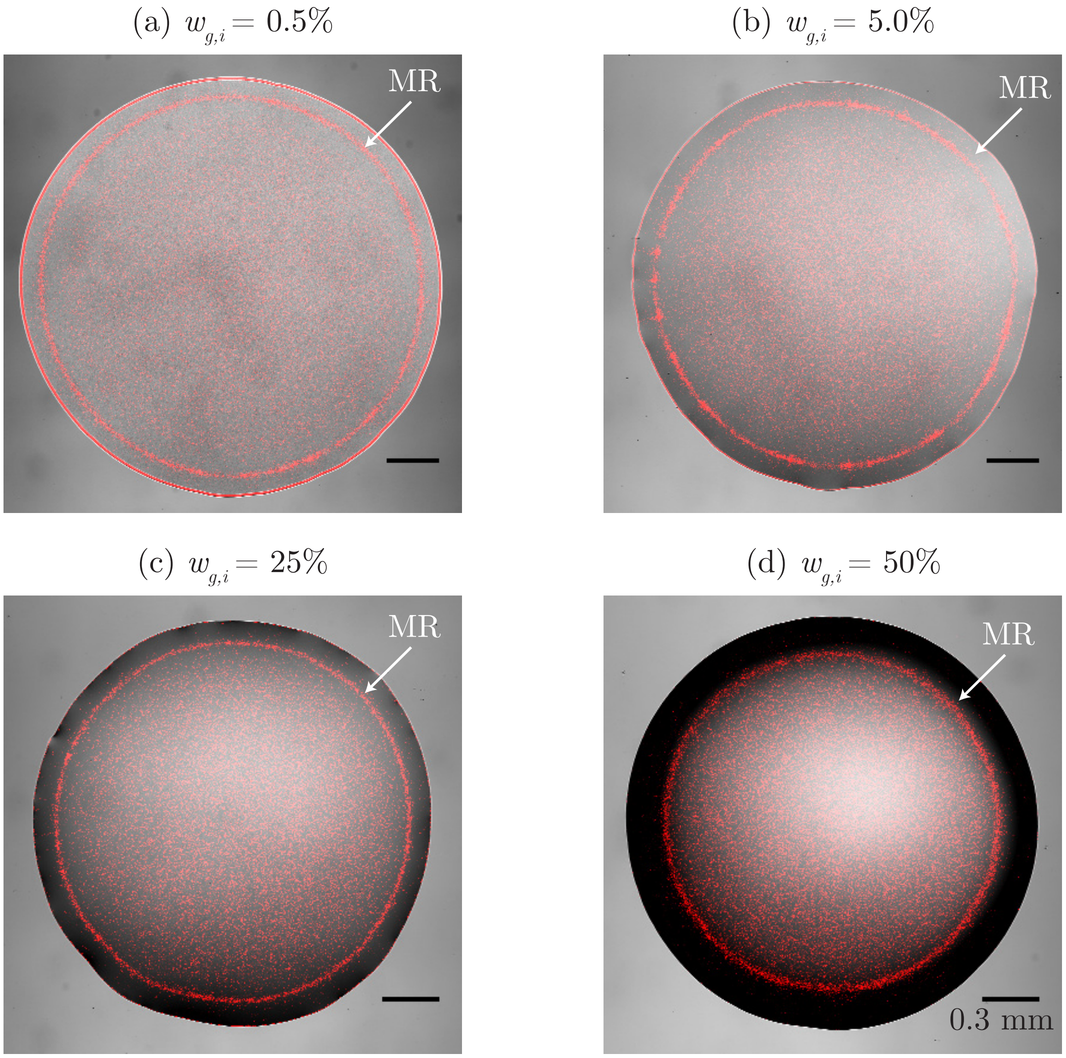

Figure S4: **Marangoni ring (MR) observed in water-glycerol droplets with initial glycerol weight fractions,$w_{g,i}$, of (a) 0.5%, (b) 5.0%, (c) 25%, and (d) 50%.** Images show the droplet 60 seconds after the initial appearance of the Marangoni ring. The figures show overlay of images obtained from transmission channel (grey-scale) and fluoroescence channel (red, showing silica particles) of the confocal microscope operated in Galavano mode. Scale bar 0.3 mm.

S6 Top-view visualization of particle-laden water droplet

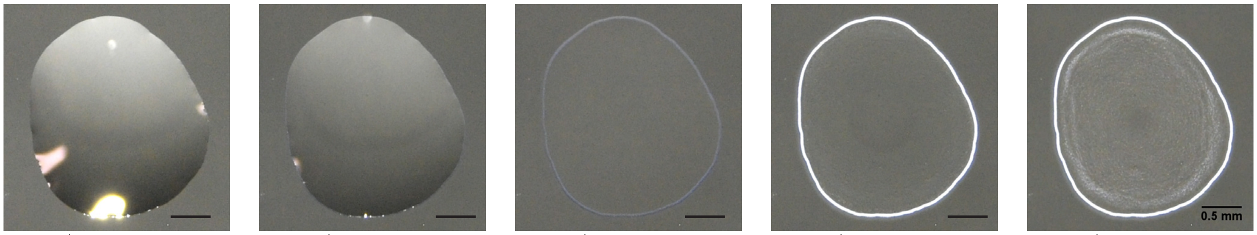

$t/t_o = 0.06$ $t/t_o = 0.39$ $t/t_o = 0.93$ $t/t_o = 0.99$ $t/t_o = 1.06$

Figure S5: **Top-view images of a water droplet containing 0.8 μm sized silica particles.** The Marangoni ring does not appear during evaporation, However, after the evaporation is complete, there is an additional ring-like deposit close to the contact line, similar to the one reported by Rossi et al.[S2] and attributed to thermal Marangoni flow. Scale bar 0.5 mm.

S7 Calculations of Rayleigh and Marangoni numbers

To compare the relative strengths of flow inside the droplet due to evaporation induced surface tension differences (Marangoni flow) versus evaporation induced buoyancy, we will estimate the Rayleigh numbers and the Marangoni numbers based on the analysis by Diddens *et al.*[S3] Rayleigh number(Ra), Grashof number (Gr), and Marangoni numbers (Ma) are defined as (cf. Eq 4.32a,b, A1, and A2 of Diddens *et al.*[S3])

$$Ra = \frac{Vg|\partial_{w_g}\rho|}{D_o\mu_o} Ev_w \tag{1}$$

$$Ma = \frac{V^{1/3}|\partial_{w_g}\sigma|}{D_o\mu_o} Ev_w \tag{2}$$

$$Gr = \frac{gh^3\rho_o(\rho_{g,pure} - \rho_{w,pure})}{\mu^2} \tag{3}$$

$$Gr = \frac{3}{\pi}\frac{1-\cos\theta}{2+\cos\theta}\frac{Ra}{Ev_w Sc} \tag{4}$$

where V is the volume of the droplet, ρ is the density of the mixture, σ is the surface tension of the mixture, h is the height of the droplet, Ev_w is a non-dimensional evaporation number, $Sc = \mu_o/\rho_o D_o$ is the Schmidt number, and D_o, ρ_o, and μ_o are the spatially averaged diffusion coefficient, mass density, and viscosity of the mixture (see Diddens et al.[S3] for more details).

Using properties of water-glycerol droplet with $w_{g,i} = 25\%$, taking $Ev_w Sc = 1000$,[S3] and $\theta = 10°$, we get $Gr = 1.35$, $Ra = 2 \times 10^5$, and $Ma = 4 \times 10^5$. Based on Figure 6 of Diddens *et al.*,[S3] for small contact angles of $\theta \approx 10°$, Marangoni flow should determine the flow pattern.

S8 Additional data on location of Marangoni ring

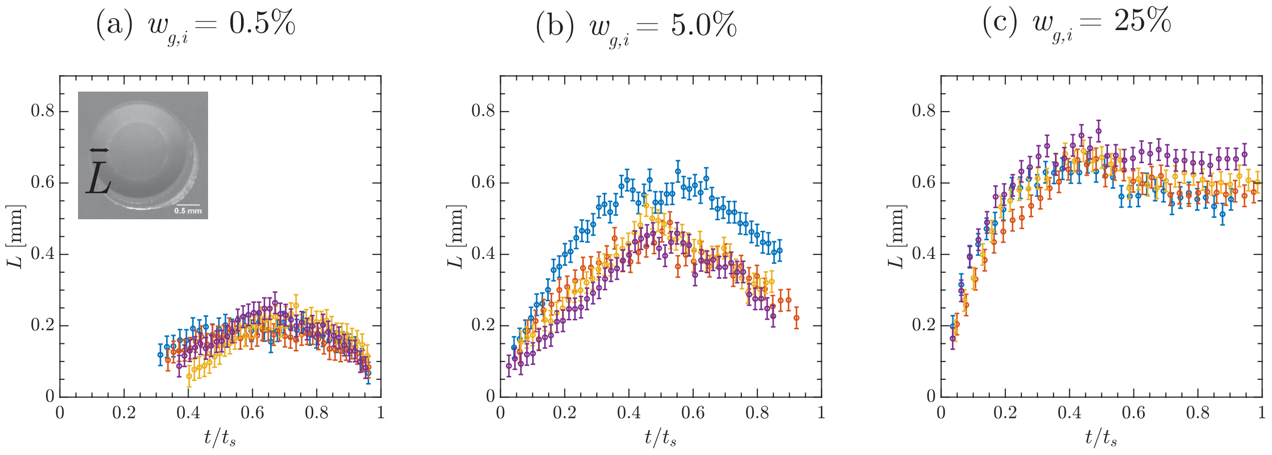

Figure S6: **Plot showing distance (L) of Marangoni ring from contact line against normalized time (t/t_s) for initial glycerol weight fractions, $w_{g,i}$, of (a) 0.5%, (b) 5.0%, and (c) 25%.** The plots show measurements from four independent experiments for each $w_{g,i}$. The errorbars are an estimate of the azimuthal variation in L at any instance.

S9 Additional data on location of Marangoni vortex

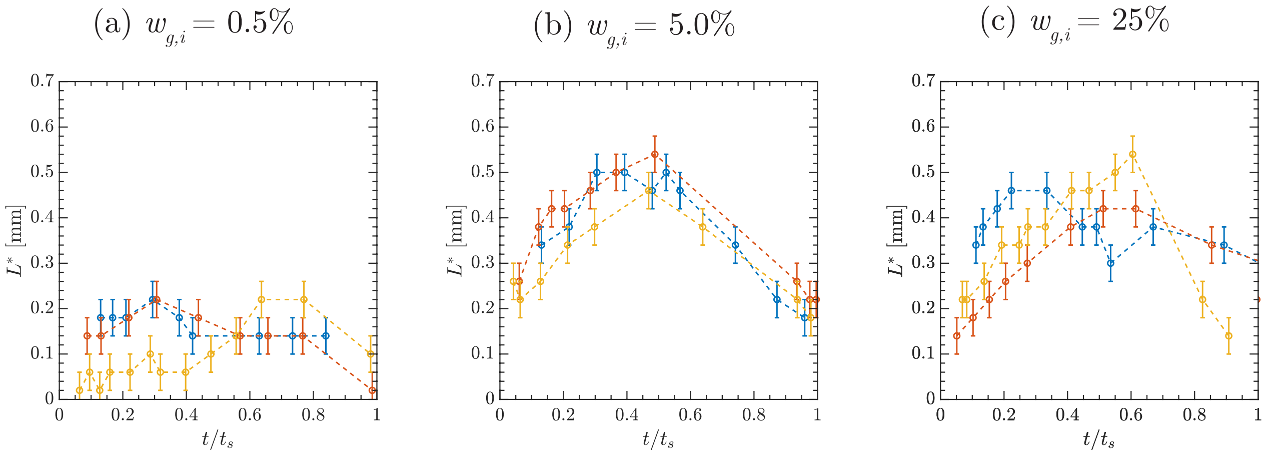

Figure S7: **Plot showing size (L^*) of Marangoni vortex from contact line against normalized time (t/t_s) for initial glycerol weight fractions, $w_{g,i}$, of (a) 0.5%, (b) 5.0%, and (c) 25%.** The plots show measurements from three independent experiments for each $w_{g,i}$. The errorbars show the uncertaininty in L^* due to the size of interrogation-area used for performing µPIV.

S10 Hypothetical influence of contaminants

Due to the severe disagreement of experimental measurements and theoretical/numerical predictions of the order of magnitude of the velocity induced by the thermal Marangoni effect in evaporating pure water droplets, it has been hypothesized that water-air surfaces are always polluted by unavoidable contaminants in experiments.[S4,S5] The presence of unknown contaminants has already been speculated in classical literature on Bénard-Marangoni convection[S6,S7] and also direct measurements of the Brownian motion of interfacial particles[S8] and the surface aging of liquid bridges[S9] came to the same conclusion. Recently, the required reduction of the surface tension due to the presence of contaminants in order to explain the differences between theory and experiments of thermal Marangoni flow in evaporating water droplets has been estimated to be on the order of 0.1 to 1 percent.[S10]

Hence, it is worthwhile to discuss the potential influence of contaminants on the Marangoni ring formation. To that end, we added a field of insoluble surfactants to the simulation. In

lack of any detailed knowledge of the properties of these contaminants, we chose a reasonable surface diffusivity of $1 \times 10^{-10}\,\mathrm{m^2/s}$ and a reduction of 0.5 % of the surface tension of pure water due to the presence of these contaminants. Therefore, we solved a transport equation for an insoluble surfactant field Γ along with the remaining equations. Due to linearity, only the product of the initial surfactant concentration Γ_0 and the surface tension reduction due to the presence of the surfactants is relevant. We therefore chose $\Gamma_0 = 1\,\mathrm{\mu mol/m^2}$ and modified the surface tension function to $\sigma(w_\mathrm{g}, \Gamma) = \sigma(w_\mathrm{g}) - \alpha\sigma(0)\Gamma/\Gamma_0$, where $\sigma(w_\mathrm{g})$ is the composition-dependent surface tension of an unpolluted water-glycerol mixture against air, $\sigma(0)$ is the surface tension of pure water and $\alpha = 0.5\,\%$ is the relative surface tension reduction of pure water due to the presence of the contaminants.

A comparison between a simulation with and without surfactants in shown in figure S8 (Also Supplementary Videos V5 and V7). Without surfactants, cf. panel (a), the solutal Marangoni flow of the glycerol-water mixture spans as a single vortex throughout the entire droplet. Thereby, the particle concentration is highest in the center of the droplet, cf. panel (c), contrarily to the experimental observations. The consideration of contaminants can counteract the Marangoni flow of the glycerol-water dynamics, which leads to a different distribution of glycerol within the droplet and, according to Raoult's law, also to an altered vapor distribution and evaporation rate of water (panel (b)). In this scenario, the Marangoni vortex is indeed limited to a finite region close to the contact line, i.e. congruent with the experimental findings. Also the particle concentration field shows a local maximum between the center and the contact line of the droplet (panel (d)).

However, due to the unknown nature of the potential contaminants, their solubility, their preference to stay e.g. on water-rich areas of the interface and any potential interactions with the particles, these results are rather speculative. Nevertheless, we conclude that neither thermal Marangoni flow, nor the consideration of gas flow dynamics were able to replicate a restriction of the Marangoni vortex to the region near the contact line in the simulations. Also, all known composition-dependent properties of glycerol-water mixtures (mass density,

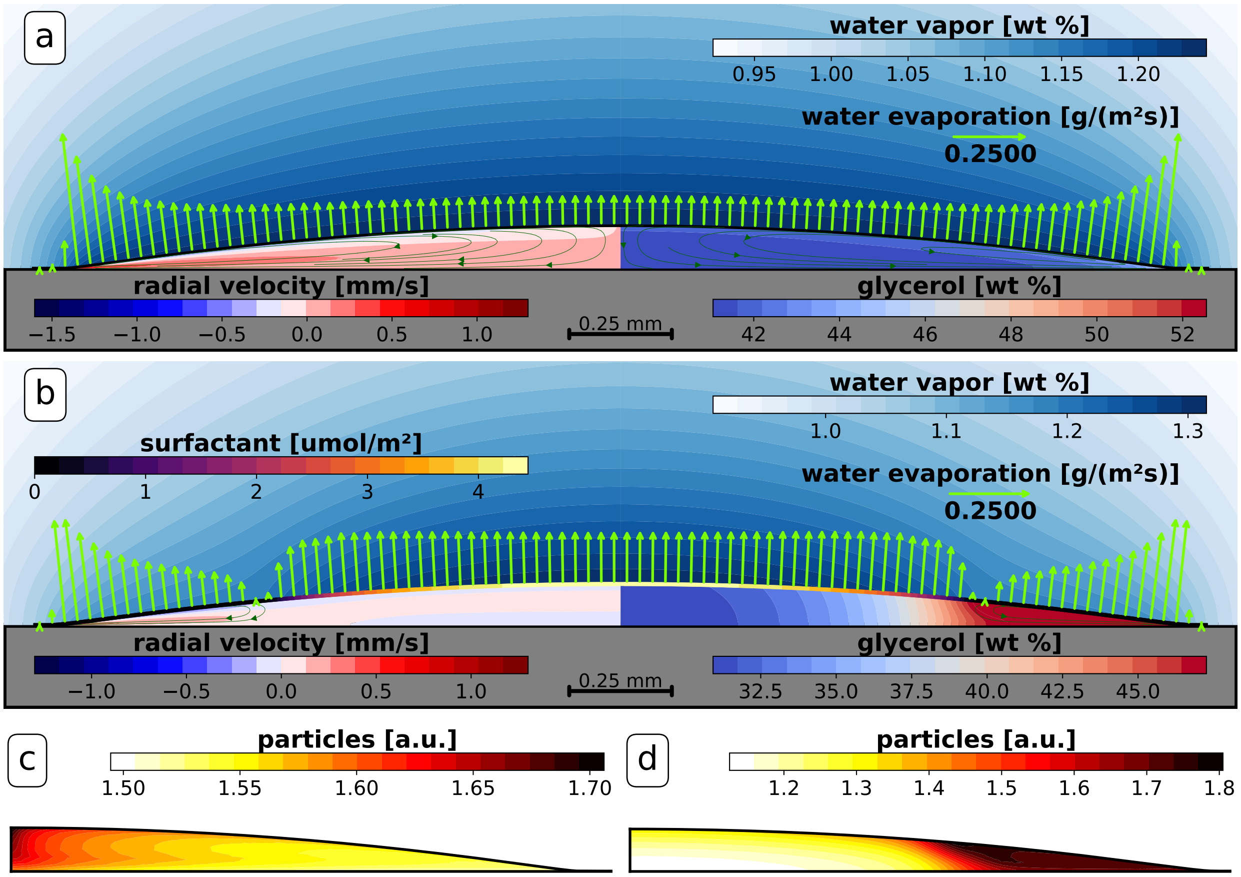

Figure S8: **Hypothetical influence of contaminants.** (a) The simulation of an uncontaminated droplet with initially 25 % glycerol shows a single Marangoni vortex throughout the entire droplet (left: radial velocity along with stream lines, right: glycerol concentration, gas phase: vapor field). (b) When surfactants are considered, the solutal Marangoni flow of the glycerol-water mixture compresses the surfactants towards the apex. This surfactant distribution (color-coded at the interface) counter-acts the Marangoni forces of the glycerol-water mixture and prevents the Marangoni flow to spread over the entire droplet. Thereby the droplet composition is altered so that the evaporation rate is also different due to Raoult's law. (c) The typical particle concentration field for the pure case (a), where most particles can be found in the central region. (d) The presence of surfactants, i.e. situation (b), leads to an enhanced particle concentration at a finite distance from the contact line, but not near the center. In both cases, the Marangoni contraction is visible, i.e. the pinned contact line has collapsed.

viscosity, surface tension, diffusivity, activity) were considered appropriately in the simulations without surfactants, but without any success to reproduce the experimental findings. Thus, a decisive influence of contaminants provides at least a reasonable scenario to explain the occurrence of the Marangoni ring.

References

(S1) Garcia, D. Robust smoothing of gridded data in one and higher dimensions with missing values. *Computational Statistics & Data Analysis* **2010**, *54*, 1167–1178.

(S2) Rossi, M.; Marin, A.; Kähler, C. J. Interfacial flows in sessile evaporating droplets of mineral water. *Phys. Rev. E* **2019**, *100*, 1–8.

(S3) Diddens, C.; Li, Y.; Lohse, D. Competing Marangoni and Rayleigh convection in evaporating binary droplets. *J. Fluid Mech.* **2021**, *914*, A23.

(S4) Cammenga, H.; Schreiber, D.; Barnes, G.; Hunter, D. On Marangoni convection during the evaporation of water. *J. Colloid Interface Sci.* **1984**, *98*, 585–586.

(S5) Hu, H.; Larson, R. G. Analysis of the effects of Marangoni stresses on the microflow in an evaporating sessile droplet. *Langmuir* **2005**, *21*, 3972–3980.

(S6) Pearson, J. On convection cells induced by surface tension. *J. Fluid Mech.* **1958**, *4*, 489–500.

(S7) Berg, J.; Acrivos, A. The effect of surface active agents on convection cells induced by surface tension. *Chem. Eng. Sci.* **1965**, *20*, 737–745.

(S8) Molaei, M.; Chisholm, N. G.; Deng, J.; Crocker, J. C.; Stebe, K. J. Interfacial Flow around Brownian Colloids. *Phys. Rev. Lett.* **2021**, *126*, 228003.

(S9) Ponce-Torres, A.; Vega, E.; Montanero, J. Effects of surface-active impurities on the liquid bridge dynamics. *Exp. Fluids* **2016**, *57*, 1–12.

(S10) van Gaalen, R.; Wijshoff, H.; Kuerten, J.; Diddens, C. Competition between thermal and surfactant-induced Marangoni flow in evaporating sessile droplets. *J. Colloid Interface Sci.* **2022**, *622*, 892–903.
